# Clinical significance of signal shadowing during intraoperative optical coherence tomography-assisted vitreoretinal surgery

**DOI:** 10.1038/s41598-024-56125-y

**Published:** 2024-03-05

**Authors:** Erick Carlos Reyna, Melisa Öztek, Goran Petrovski, Susanne Binder, Knut Stieger, Lyubomyr Lytvynchuk

**Affiliations:** 1 Department of Ophthalmology, Eye Clinic, Justus Liebig University, University Hospital Giessen and Marburg, Campus Giessen, Giessen, Germany; 2 Center for Eye Research and Innovative Diagnostics, Department of Ophthalmology, Institute for Clinical Medicine, University of Oslo, Oslo, Norway; 3 Department of Ophthalmology, Oslo University Hospital, Oslo, Norway; 4 Department of Ophthalmology, University of Split School of Medicine and University Hospital Centre, Split, Croatia; 5 UKLONetwork, University St. Kliment Ohridski-Bitola, Bitola, North Macedonia; 6 Karl Landsteiner Institute for Retinal Research and Imaging, Vienna, Austria; 7 Eye Center Donaustadt, Sigmund Freud University, Vienna, Austria

**Keywords:** Retinal diseases, Biomarkers

## Abstract

This study aimed to analyze the clinical significance of signal shadowing during intraoperative optical coherence tomography (iOCT)-assisted vitreoretinal surgery caused by vitreoretinal instruments, tissue dyes, and vitreous substitutes, and to objectively quantify its impact on iOCT imaging. This is a retrospective observational study of postoperative image analysis from one hundred seventeen (117) patients who underwent iOCT-assisted vitrectomy. The image data were divided into three groups: vitreoretinal instruments, tissue dyes, and vitreous substitutes. The data was then processed using graphic software to measure the grade of picture quality distortion and compared to paired image controls without clinically perceptive interference, then analyzed statistically. The intraocular portion of all studied vitreoretinal instruments caused a high average gray level interference compared to controls ranging from 32 to 68% reduction, obscuring the area of interest significantly. The tips of the instruments produced low-grade shadowing, allowing the underlying tissue to be distinguished. The analyzed dyes demonstrated a wide interference range: ICG (− 75.12%), and triamcinolone (− 26.13%) showed dose-dependent high shadowing, while VITREODYNE™ (49.3%) and brilliant blue G (14.06%) exhibited no perceived distortions whilst increasing average gray levels. All analyzed vitreous substitutes (air, SF_6_, C_3_F_8_, PFCL, and silicone oil) showed an insignificant shadowing effect on iOCT. Certain dyes and vitreous substitutes produce a negligible shadowing effect compared to controls and other dyes, providing an advantage during real-time iOCT imaging. All analyzed vitreoretinal instruments showed a significant interference that should prompt the development of new imaging techniques or the implementation of materials with low-grade interference to overcome a clinically relevant shadowing effect on iOCT, maximizing the technology’s visual accuracy and surgical diagnostic aid proficiency.

## Introduction

Optical coherence tomography (OCT) is a non-invasive imaging method used in ophthalmology that renders images of the eye comparable to histologic examinations^8,23^. The OCT camera projects low-coherence light towards the object being analyzed to subsequently record the scatter pattern and intensity of the light being reflected to build a high-quality cross-sectional image. This concept, known as interferometry, functions comparatively to an ultrasound, but based on light waves instead^8,9^. These characteristics enable OCT to accurately visualize translucent objects^17^.

Due to these features, just like the pioneering time domain OCT, the currently used spectral domain and swept source OCTs rapidly became a central diagnostic tool in ophthalmology, redefining the assessment criteria for many ophthalmologic disorders^1^. While employed on both the anterior and posterior segments of the eye, OCT imaging is particularly useful to detect and monitor retinal pathologies^14,17^. Retinal anatomy, especially its layered structure has been extensively documented using OCT, providing a concrete visualization of cellular layers. This has impacted the way follow-up assessments are carried out before and after therapeutic interventions, and how treatment efficacy is measured in retinal diseases^4^.

As novel uses of OCT technology within ophthalmology continue to grow exponentially, the technology’s integration with surgical microscopes has improved the development of intraoperative OCT (iOCT), enabling real-time microscopic tissue examination during surgical procedures without interruption^5^, and even developing new prospective applications in the fields of retinal prosthetics and gene therapy^26,28^. Specifically, during vitreoretinal surgery, iOCT creates an additional perspective to the microscope’s traditional downward-scope view^13^. Real-time tissue analysis during surgical procedures has provided new information about the pathophysiology of certain eye diseases and intraoperative tissue behavior^10,24^. At the same time, new challenges arise as this imaging technique is being introduced into diagnostic and treatment schemes. One of these problems has been the visual interference or shadowing caused by the numerous instruments, contrasting substances, and diverse vitreous substitutes used intraocularly during vitrectomy, reducing the quality of iOCT images, hence reducing the visualization of the area of most interest and diminishing its scope of use^30^. This problem has been especially limiting to surgical maneuvers in the macular area, hindering the reliability of iOCT during membrane removal, subretinal injections of medications and closure of macular holes^12,15^.

The degree to which this technology and its application are limited by this phenomenon has, until now, only been determined clinically by the operating surgeon. Objective markers to measure such visual challenges have not been widely documented^11,13^. In recent years, multiple experimental research projects regarding iOCT have been published, exhibiting a wide array of user-specific approaches to iOCT applications within the different ophthalmic subspecialties^15,17,18^, as well as in other medical specialties^25^. Standardization attempts are still ongoing^6^.

The purpose of this study is to analyze the significance of signal shadowing during iOCT-assisted vitreoretinal surgeries caused by vitreoretinal instruments, dyes, and vitreous substitutes, and to objectively quantify its impact on iOCT imaging. To our knowledge, this is the first systemic study of signal shadowing during intraoperative optical coherence tomography-assisted vitreoretinal surgery.

## Methods

### Ethical statement

This retrospective non-randomized observational consecutive case series study was performed at the Department of Ophthalmology at Justus Liebig University, Eye Clinic, University Hospital Giessen and Marburg GmbH, Campus Giessen, Germany between 2019 and 2023. The study has followed the tenets of the Declaration of Helsinki and was approved and registered by the onsite Ethics Commission of the Department of Medicine at Justus Liebig University in Giessen. All patients were informed about the surgical treatment protocol regardless of their disease and bestowed their signed informed consent before the procedures. All diagnostic measures and therapeutical treatments undertaken were performed following good clinical practices and current local and international medical guidelines and regulations.

### Patients

Imaging data from one hundred seventeen (117) patients undergoing an iOCT-assisted vitrectomy was analyzed; 63% of the patients were men, 45% were over 60 years of age, and for 14 patients the procedure performed was reoperation. The diagnoses that prompted operative treatment were epiretinal membranes (n = 52), macular holes (n = 41), retinal detachment (n = 27), and persistent vitreous hemorrhage (n = 8); overlapping diagnosis occurred in a few cases, particularly with concomitant macular holes and epiretinal membranes.

### Surgical procedures and data collection

All patients underwent *pars plana* vitrectomy using 23-, 25- or 27-gauge trocars to access the posterior segment of the eye. Depending on the underlying pathology being treated, a series of multiple instruments, dyes, and vitreous substitutes were employed accordingly. Video recordings from each procedure were obtained using an ophthalmologic surgery microscope Rescan® 700 (Carl Zeiss Meditech, Oberkochen, Germany), which includes a fully integrated iOCT system with the following technical characteristics: spectral domain OCT source, wavelength 840 nm, scanning speed 27,000 A-scans/second, axial resolution 5.5 μm, refresh rate 5 Hz-50 Hz, scan depth 2.5 mm, scan length 9 mm (range: 3–16 mm), scan mode cross-hair. During surgeries, iOCT video and snapshot data with and without a specific instrument, dye, or vitreous substitute were recorded. Through intraoperative scenarios, iOCT imaging was directly interpreted by the operating surgeon and assistant surgeon. Postoperatively, images were analyzed by the entire research group (two graders) who routinely study iOCT images, and who determined by consensus the presence of clinically perceived shadowing. This criterion was later compared to statistically significant measurable shadowing.

### Groups

The iOCT images of these vitreoretinal procedures were divided into three main groups depending on the material being analyzed: (1) vitreoretinal instruments, (2) tissue dyes, and (3) vitreous substitutes. Each category was comprised of the following analyzed items: (1) vitreoretinal instruments: vitrectome, ILM forceps, silicone tipped cannula (D.O.R.C. International BV, Zuidland, Netherland), Finesse® Flex Loop (Alcon Laboratories Inc., Fort-Worth, TX, USA), and Tano Diamond Dusted Membrane Scraper (Synergetics®, Bausch&Lomb, Rochester, NY, USA); (2) stains: indocyanine green, brilliant blue G ophthalmic (D.O.R.C. International BV, Zuidland, The Netherlands), triamcinolone acetonide, and a lutein-based dye VITREODYNE™ (Kemin Pharma, Barcarena, Portugal); (3) vitreous substitutes: air, sulfur hexafluoride (SF_6_), octafluoropropane (C_3_F_8_), perfluorocarbon liquid (PFCL) (D.O.R.C. International BV, Zuidland, Netherland), and silicone oil.

### Post-processing and statistical analysis

The iOCT video and snapshot data was post-processed using graphic software (ImageJ, LOCI, University of Wisconsin, USA) to measure the grade of picture quality distortion through a histogram analysis measuring average gray level (GL) interference and compared with paired image controls without clinically perceptive interference. GL images consisted of pixels exhibiting different brightness or intensity levels, forming a grayscale depiction. In this type of images, the brightness or GL is expressed through numerical values at specific intervals, with a wider range of shades in the image corresponding to a larger interval. A higher level expresses a higher intensity, while a lower level corresponds to a lower intensity^16^. iOCT video series of retinal tissue scans without perceivable signal blocks were analyzed to determine a GL control value. These were then compared to the GL measured from paired recording sequences, where shadowing caused by a study material was present over the iOCT scan, as it would during standard vitrectomy. To our knowledge, no standardized image quality measurement, quantifying the level of shadowing effect during iOCT scans has been previously established. This was done by analyzing multiple square 100 × 100 pixel picture frames within the collected recording sequences of each group item and their paired controls. When selecting images for the histogram analysis from within the original iOCT video recordings, only picture frames with one type of study material being visible within the scope of the iOCT scan were chosen; therefore, filtering out potential confounding shadowing effects from other materials. Individually sized, smaller pixel frames were utilized in few instances where aberrant image artifacts caused by certain intraocular tools distorted the GL analysis. Images with lower positive pixel counts recorded a lower GL on the histogram analysis, hence exhibiting a higher level of interference. The data was statistically analyzed using spreadsheet software (Microsoft Excel). The dataset analyzed during the current study can be made available from the corresponding author upon request.

## Results

**Figure 1:**
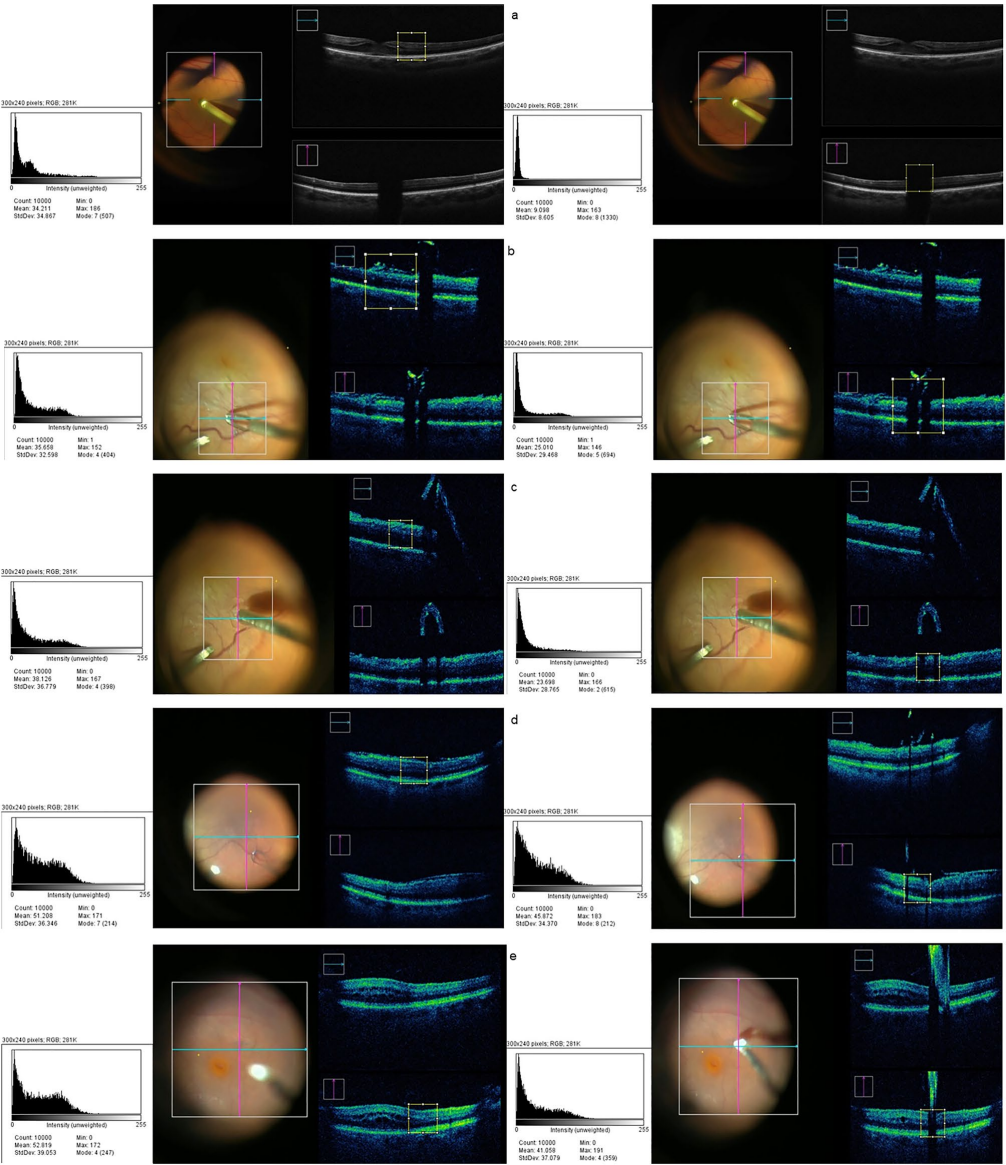
Histogram analysis comparison examples between controls (left) and study images (right) with noticeable shadowing effect of the analyzed tools: (**a**) Vitrectome. (**b**) ILM forceps. (**c**) Silicone tipped cannula. (**d**) Finesse® Flex Loop. (**e**) Tano Diamond Dusted Membrane Scraper.

After adjusting for shadow size and image artifacts, the intraocular portion of all vitreoretinal instruments caused a high average GL interference compared to controls. Specifically, the vitrectome exhibited an extreme shadowing effect with a 68.51% GL reduction in the study area. Similarly, ILM forceps showed 36.21% lower GL (25.47 ± 7.34 vs. 39.94 ± 6.57), and silicone tipped cannula 43.96% lower GL (22.89 ± 6.01 vs. 40.85 ± 2.26). ILM forceps, cannulas, and vitrectomes cast large and dense shadows, covering the underlying tissue being analyzed completely. Instruments with smaller tips, such as Finesse® Flex Loop, produced a low-grade shadowing, allowing the underlying tissue to be distinguished. Other instruments consistently created hyperreflectivity artifacts during iOCT recordings that produced a similar or higher GL compared to controls when analyzed. This occurred with the Tano Diamond Dusted Membrane Scraper (5.51% GL increase; 44.04 ± 4.72 vs. 41.74 ± 6.4) and Finesse® Flex Loop (13.75% GL increase; 51.43 ± 2.86 vs. 45.21 ± 0.65). Smaller shadow-specific pixel frames accounting for this phenomenon were measured in such cases and showed a stark shadow effect, reducing the perceived GL similarly to other vitreoretinal instruments as shown in Table 1. Nevertheless, in these cases, the clinically perceived shadowing effect was minimal to mild. Control and study image examples of the analyzed vitreoretinal tools illustrating the GL comparison performed through histogram analysis can be seen in Fig. 1.

**Table 1:** Gray level of different vitreoretinal surgery instruments.

Instrument	Mean gray level	Standard deviation	Offset from control	p-value (significance < 0.05)
Vitrectome	12.57	2.05	− 68.51%	< 0.001
ILM forceps	25.47	7.34	− 36.21%	< 0.001
Silicone tipped cannula	22.89	6.01	− 43.96%	< 0.001
Finesse® flex loop	51.43	2.86	13.75%	< 0.001
Finesse® flex loop (shadow adjusted)	30.4	7.2	− 32.75%	< 0.001
Tano diamond dusted membrane scraper	44.04	4.72	5.51%	< 0.001
Tano diamond dusted membrane scraper (shadow adjusted)	18.43	9.48	− 55.84%	< 0.001

**Figure 2:**
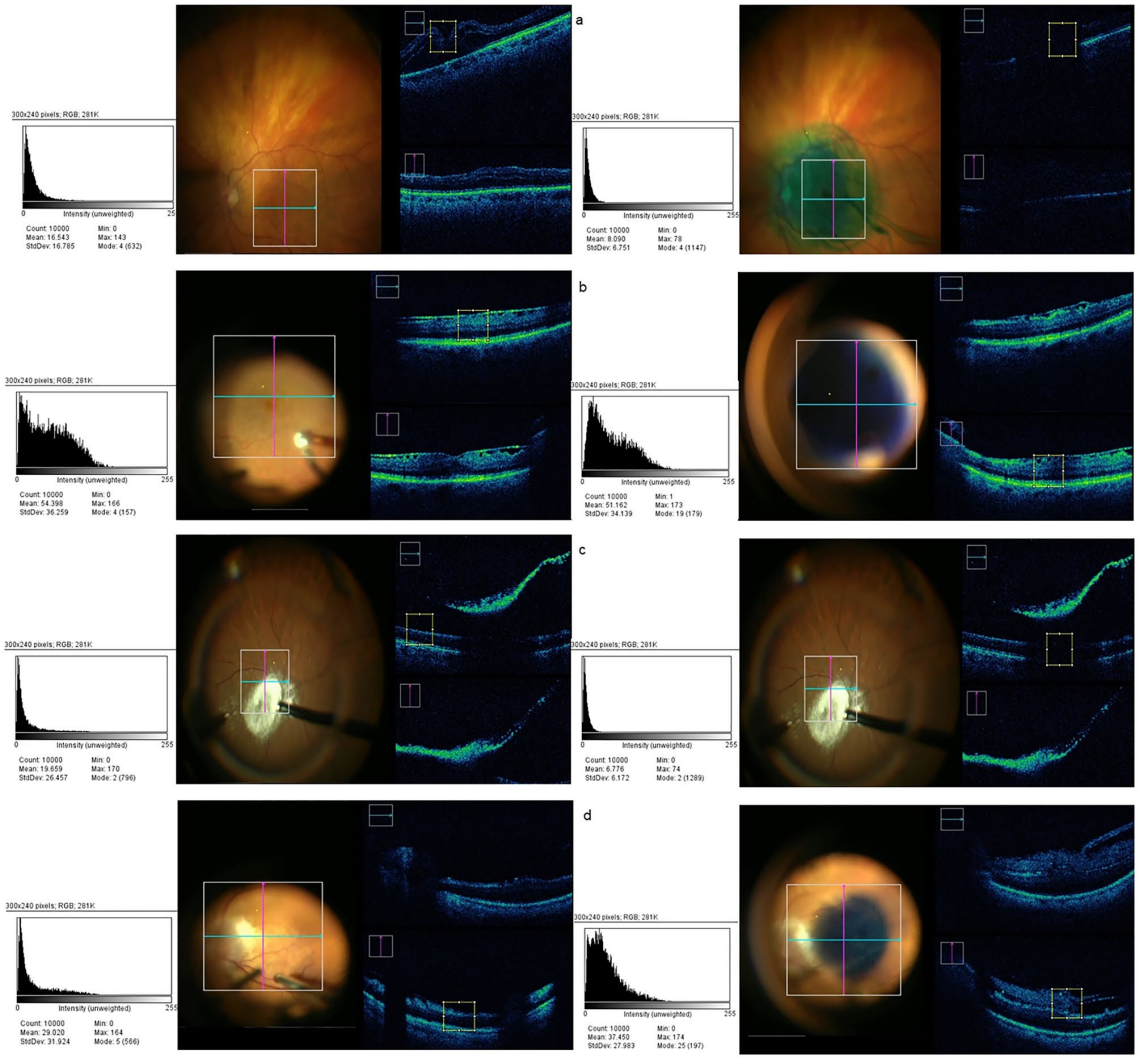
Histogram analysis comparison examples between controls (left) and study images (right) of the analyzed stains: (**a**) Indocyanine green. (**b**) Brilliant blue G. (**c**) Triamcinolone acetonide. (**d**) Lutein-based dye VITREODYNE™.

The analyzed dyes demonstrated a wide range of image interference patterns on iOCT, as depicted in Table 2. Indocyanine green (75.12% lower GL; 8.34 ± 1.37 vs. 33.52 ± 10.48) and triamcinolone acetonide (26.13% lower GL; 13.9 ± 10.0 vs. 18.81 ± 0.84) showed a medium to high shadowing effect of the underlying tissue when compared to controls. Triamcinolone acetonide particularly demonstrated a dose-dependent interference effect, with more substance resulting in more interference, which directly correlates to the high standard deviation observed. On average, the lutein-based dye VITREODYNE™ demonstrated a significantly higher GL (49.3%; 31.84 ± 8.37 vs. 21.32 ± 5.16) when compared to controls because the substance itself was detected by the iOCT camera while casting no relevant shadow over the underlying tissue. A similar effect with no visually perceptible interference was observed with brilliant blue G, but with only a marginally higher GL (14.06%) as compared to controls (47.75 ± 8.96 vs. 41.86 ± 7.05), exhibiting no significant distortion. Control and study image examples of the analyzed stains illustrating the GL comparison performed through histogram analysis can be seen in Fig. 2.

**Table 2:** Gray level of different tissue dyes.

Dye	Mean gray level	Standard deviation	Offset from control	p-value (significance < 0.05)
Indocyanine green	8.34	1.37	− 75.12%	< 0.001
Brilliant blue G	47.75	8.96	14.06%	< 0.001
Triamcinolone acetonide	13.9	10.0	− 2.61%	< 0.001
Lutein-based dye VITREODYNE™	31.84	8.37	49.3%	< 0.001

**Figure 3:**
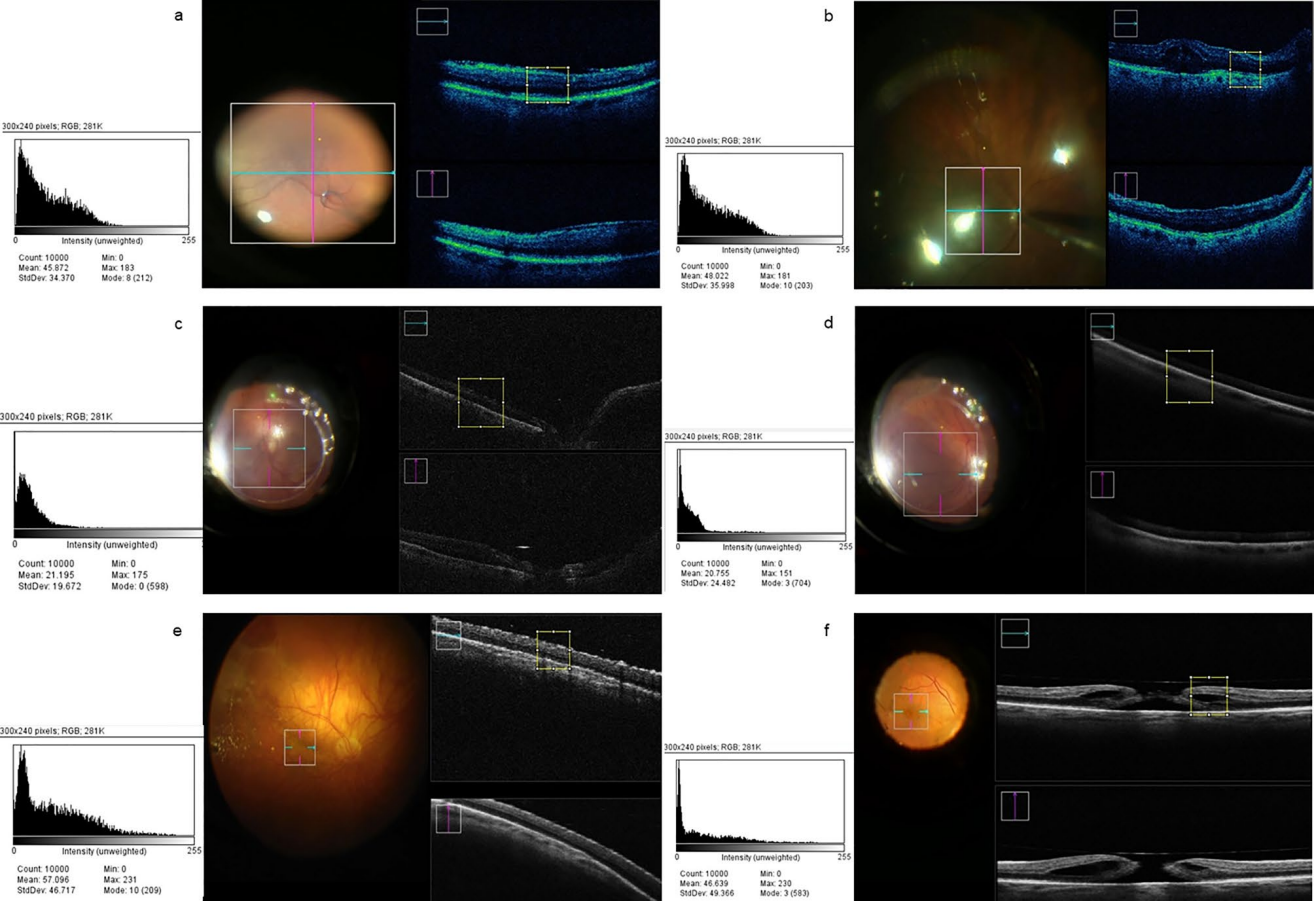
Histogram analysis examples of the vitreous substitutes analyzed: (**a**) BSS (control). (**b**) Air. (**c**) SF_6_. (**d**) C_3_F_8_. (**e**) PFCL. (**f**) Silicone oil.

All clinically analyzed vitreous substitutes showed an insignificant to minimal shadowing effect on iOCT. When compared objectively to balanced salt solutions (BSS) as controls, this phenomenon was generally confirmed: air − 0.29% (p-value: 0.97), SF_6_ − 2.6% (p-value: 0.77), C_3_F_8_ − 5.52% (p-value: 0.56), PFCL 1.6% (p-value: 0.83) and silicone oil 9.24% (p-value: 0.32), as exhibited in Table 3. In general, gases tended to produce a reduction of GL, while highly viscous liquids produced a relatively higher GL. In both cases, the observed offset from controls was minimal and not statistically significant. Control and study image examples of the analyzed vitreous substitutes illustrating the GL comparison performed through histogram analysis can be seen in Fig. 3.

**Table 3:** Gray level of different vitreous substitutes.

Vitreous substitute	Mean gray level	Standard deviation	Offset from control	p-value (significance < 0.05)
Air	39.82	5.7	− 0.29%	0.97
SF_6_	20.77	4.92	− 2.6%	0.77
C_3_F_8_	20.15	0.77	− 5.52%	0.56
PFCL	40.58	4.8	1.6%	0.83
Silicone oil	43.63	2.21	9.24%	0.32

## Discussion

This study aimed to examine the clinical significance of the signal shadowing during iOCT-assisted vitreoretinal surgery focusing on the signal shadowing created by vitreoretinal instruments, tissue dyes, and vitreous substitutes, which reduces the visualization of the area of most interest. One hundred and seventeen eyes were subjected to a vitrectomy and recorded using iOCT. During these procedures, a wide array of vitreoretinal instrumentation, dyes, and vitreous substitutes were utilized. Data post-processing was performed by graphic software to determine the average GL through a histogram analysis. Paired image controls of each sequence were also analyzed. This study was carried out in a tertiary-level university hospital with a high volume of cases. To our knowledge, no previous study has quantified and statistically examined these phenomena. The interpretation of the statistical comparative analysis of the study images and the controls delivered the following generalizations: (1) clear materials create an insignificant or very low-grade shadowing effect; (2) material density is intrinsically correlated to the shadowing effect produced, whereby less concentrated materials generally produce a lower level of shadowing and highly concentrated materials exhibit more shadowing; (3) small items, irrespective of their density, produce clinically irrelevant shadowing due to the small size of the shadows they cast over the analyzed areas. Nevertheless, this assessment cannot be applied in each case, given that several cases exhibited complex interference behavior, revealing distinct variables that play a role in the shadowing effect registered on iOCT.

Considering that all analyzed instruments produced an objectively high reduction of GL when adjusting for shadow size and image artifacts, it can be deduced that these variables have a clinical implication on the perceived interference when compared to the measured interference. Perhaps this can be best exemplified by the following findings: larger items with dense metallic hulls produced high interference both perceptively and objectively, which was the case with most cannulas and ILM forceps studied. On the other hand, in the case of silicone tipped cannulas and Finesse® Flex Loop, the tips of the instruments visually produced thin low-grade shadowing, allowing the underlying tissue to be distinguished on iOCT. Nonetheless, size-specific objective measurements of the shadowed areas rendered a high gray level reduction as compared to paired controls. In these cases, the shadow areas were always smaller than a 50 × 50 pixel field, which is half the size of the standard area used during the analysis. This small dimension was interpreted by our study group to be clinically irrelevant because the outline of the underlying tissue could still be easily identified^20^. This was also the approach taken by several studies that developed similar analytical interpretations or adopted such benchmarks in their study design^2,7^. Yet, this discrepancy prompts the need to establish objective criteria that determine what a clinically relevant interference is, to create a standard unitary interpretation that could ensure analysis reproductivity and enhance the compatibility of future references of the data. Similar questions have been indirectly posed by other studies^12,19^.

In some other instances, vitreoretinal instruments and stains have rendered hyperreflective artifacts on iOCT recordings that simultaneously spiked the measured GL on histographic analysis and obscured underlying tissue visualizations. This prompted a new shadow-specific analysis to obtain a more adequate representation of the interference. Identical behavioral results of the shadow-specific analysis were found: size-specific pixel analysis rendered significant GL reduction that was nonetheless only deemed to be clinically relevant when the recorded artifact size caused by the material used was large enough to obscure the underlying tissue. Similar issues were also observed in previous studies^21^.

The investigated dyes displayed a range of interference behaviors on iOCT. Indocyanine green and triamcinolone acetonide exhibited high to medium shadowing effects on the underlying tissue compared to controls, which increased sharply as the substance concentration increased. Notably, triamcinolone demonstrated more of a dose-dependent interference behavior, whereby greater substance concentration resulted in more pronounced interference, as reflected by its relatively high standard deviation (10.0) but a small offset from controls (− 2.61%); in these instances, a gradual GL reduction was observed, which correlated with the clinically perceived interference, even though the intensity of the casted shadow created a statistically significant (p-value: < 0.001) GL reduction when adjusted for shadow size and hyperreflectivity artifacts, even with minimal substance concentrations. VITREODYNE™, the lutein-based dye, showed significantly higher gray levels as compared to controls, given that the substance itself was detected by the iOCT camera, but without causing significant shadowing, neither measured nor perceived. This property can probably be traced back to the product’s relatively high infrared light transparency^22,27^. Brilliant blue G, which has similar physical properties, exhibited no visually perceptible interference and only slightly higher GL than controls, indicating no significant distortion.

In terms of vitreous substitutes, the analysis demonstrated an insignificant shadowing effect on iOCT. Both air and PFCL exhibited comparable GL when compared to controls, indicating minimal interference. These findings suggest that vitreous substitutes have a limited impact on iOCT imaging, thereby maintaining clarity and enabling effective visualization during vitreoretinal surgeries. Gases tended to produce a reduction of GL, while highly viscous liquids produced a relatively higher GL. This phenomenon correlates with the perceived distortion, which was in every case minimal^3^.

These findings support the conclusion that, apart from size and transparency, the main characteristics that determine the shadow-generating effect on iOCT of an instrument appear to be the physical state of the interfering item and the material density or concentration. Other physical properties such as polarization, fluid-lipid affinity, and refractive index are variables that could further aid in understanding which materials are better suited to be used in clinical settings during iOCT-assisted vitrectomies. Naturally, the camera system must be taken into account to appropriately anticipate image distortion caused by different types of signal interrupters^29^. Considering these elements, materials used intraocularly during vitreoretinal procedures should ideally produce little to no shadow effect. If unavoidable, thin instrumentation that produces only partial interference should be preferred. These findings demonstrate the benefit of thin-gauged instruments during vitreoretinal surgery assisted with iOCT.

One of the limitations of this could be the fact, that our samples were taken using a single type of ophthalmologic surgical microscope—Rescan® 700 (Carl Zeiss Meditech, Oberkochen, Germany), coupled with house-brand ZEISS CALLISTO eye® iOCT software. Therefore, slight differences could potentially arise when compared to samples taken using microscopes with different iOCT software versions. However, the signal shadowing relations between the control and study groups appeared to be visible and significant. Even though a thorough selection of the analyzed images was performed, ruling out frames where evidently more than one study item was present, a completely independent scan cannot be unequivocally ascertained, since all stains and many vitreous substitutes are delivered or manipulated intraocularly in real-time with cannulas and vitrectomes. This limitation could possibly misconstrue the origin of the shadow, misrepresenting the intensity of the shadowing effect exhibited by each material studied. Nevertheless, to minimize this possible confounding factor, our study group only selected images where one type of material was visible within the scope of the iOCT scan for the analysis, therefore filtering out potential confounding shadowing effects from other materials.

Another limitation of this study could be the use of the selected instruments, tissue dyes, and vitreous substitutes produced by certain companies. The use of similar products made by alternative companies could influence the results of the signal shadowing analysis.

All images consisted of B-scan images; different field appearances were due to slight setting variations as per surgeon’s preference. Similarly, we registered four iOCT video sequences with macular edema and two with macular holes where a slight offset between the control and study area could be found, mainly due to shorter video sequences over the same retinal area during iOCT recordings. Upon revision the best possible comparable areas were selected, though slight offsets of the same anatomical area of the retina could imply different base GLs in these cases. Nevertheless, every analyzed shadow caused by an item was paired with a control image without perceivable shadowing from the same scan series within the same retinal quadrant, effectively having the same visual settings; this allowed objective comparison accordingly.

## Conclusions

This study aimed to examine the clinical significance of signal shadowing during iOCT-assisted vitreoretinal surgeries caused by vitreoretinal instruments, dyes, and vitreous substitutes, and to quantitatively assess its influence on iOCT imaging. To our knowledge, this is the first systemic analysis of this kind. The findings provide valuable insights regarding the significance of different factors contributing to image interference and shadowing effects. Overall, this study sheds light on the importance of considering signal shadowing in iOCT-assisted vitreoretinal surgeries. The results emphasize the varying degrees of interference caused by different vitreoretinal instruments and stains, while also highlighting the negligible impact of vitreous substitutes. These insights can aid surgeons in making decisions regarding instrument selection and stain usage, ultimately enhancing the accuracy and efficacy of iOCT-guided procedures in vitreoretinal surgery in order to keep good visibility of the area of interest during the entire surgery. Future research could further explore strategies, such as the development of new instrumentation, to minimize signal shadowing and optimize iOCT imaging to maximize its potential for improved surgical outcomes.
